# On the Nature of the Bonding in Coinage Metal Halides

**DOI:** 10.3390/molecules27020490

**Published:** 2022-01-13

**Authors:** Slađana Đorđević, Slavko Radenković, Sason Shaik, Benoît Braïda

**Affiliations:** 1 Faculty of Science, University of Kragujevac, 34000 Kragujevac, Serbia; sladjadj93@gmail.com; 2 Institute of Chemistry, Hebrew University of Jerusalem, Jerusalem 91904, Israel; sason@yfaat.ch.huji.ac.il or; 3 Laboratoire de Chimie Théorique, Sorbonne Université, UMR7616 CNRS, 75005 Paris, France

**Keywords:** coinage metals, transition metal halides, valence bond, chemical bonding, charge-shift bonds

## Abstract

This article analyzes the nature of the chemical bond in coinage metal halides using high-level ab initio Valence Bond (VB) theory. It is shown that these bonds display a large Charge-Shift Bonding character, which is traced back to the large Pauli pressure arising from the interaction between the bond pair with the filled semicore d shell of the metal. The gold-halide bonds turn out to be pure Charge-Shift Bonds (CSBs), while the copper halides are polar-covalent bonds and silver halides borderline cases. Among the different halogens, the largest CSB character is found for fluorine, which experiences the largest Pauli pressure from its σ lone pair. Additionally, all these bonds display a secondary but non-negligible π bonding character, which is also quantified in the VB calculations.

## 1. Introduction

Understanding the nature of bonds in transition metal-containing molecules is a more challenging task than for main group atoms [1,2,3,4,5,6,7]. A key reason is the presence of two different valence shells, the (*n*−1)d and *n*s shells, which have similar spatial extensions and energies. These orbitals can be partially filled depending on the metal and its oxidation state and will usually involve a larger number of valence electrons than main group elements, which represent an additional complexity [8]. Another important characteristic of transition-metal chemistry is the importance of relativistic effects, which shrink the size of the valence s orbital and lead to the expansion of the inner d orbitals [8,9]. In the series of the coinage metals (Cu, Ag, and Au), for instance, the relativistic effects increase further down a given column of the periodic table, reaching a paramount effect for gold.

All these particularities lead to substantial correlation effects on transition metal bonding, often involving a combination of different types of correlation which in return require an adequate and elaborate theoretical treatment. With a few notable exceptions [2,6,7,10], ab initio classical Valence Bond (VB) theory [11,12,13,14] has not dealt with this important area of chemistry. From their intimate connection with Lewis structures and the electron pair bond concept, classical VB wave functions can provide intuitive and detailed insight into the nature of chemical bonding. They can also be fruitfully combined with complementary interpretative approaches such as, for instance, the “Atom-in-Molecules” (AIM) method [15]. We shall also mention empirical VB methods, which have been widely applied to understand, in particular, enzymatic reactions [16,17,18]. Previous VB studies [6,7] have shown that metal–metal bonding, including the bonding between coinage metals, exhibited a significant “Charge-Shift Bonding” (CSB) [19,20,21,22,23], which is a distinct class of bonding alongside the traditional covalent and ionic bond families. Bonding in CSB does not originate, as in covalent bonds, from the spin pairing of the covalent structure, or, as in ionic bonds, from the electrostatic stabilization of the ionic structure, but predominantly from the resonance energy arising from the covalent-ionic mixing. CSB is typically prominent in situations where the bond in question is surrounded by other electron pairs which apply strong Pauli “pressure” on the bond in question, and thereby weaken the covalent component of the bond, a simple prototype being the F-F molecule. In addition, CSB [21,22,23] has been shown to be the main bonding mechanism responsible for the stability of the inverted bond in propellane [24], two-center three-electron bonding [25], and electron-rich hypervalent systems [20]. In the case of the previously studied metal–metal bonds [6,7], CSB was found to originate from the Pauli repulsion between the bonding electrons and the semicore d shell, which, depending on the metal atom and its oxidation state, can be filled to different extents.

The present paper applies classical VB theory to a series of coinage metal halides MX (M = Cu, Ag, Au; X = F, Cl, Br). In the past few decades, these molecules have been the focus of both experimental and theoretical investigations, mainly because of their extensive applications, ranging from the traditional use of silver bromides in photography [26] and holography [27] to more recent uses of copper halides as catalysts [28] and gold halides as the building blocks of promising solid materials [29]. Besides, various transition metal complexes featuring analogous coinage metal halogen bonds have recently emerged as potential selective anticancer drugs [30,31]. As such, understanding the nature of the bonding in coinage metal halides is of fundamental importance. Generally, most of the coinage metal halides are considered to have ionic bonding characteristics, while more significant covalent bonding characteristics have been attributed to some gold halides [32,33,34,35]. Despite the usefulness of the coinage metal halides, the possibility of the occurrence of CSB in these compounds has not been studied so far. It is reasonable to expect that the presence of an electron-rich coinage metal M containing a filled (*n*−1)d semicore shell acting as lone pairs, together with a halogen X bearing lone pairs, can cause a significant CSB characteristics in the resulting MX dimers. To tackle this bonding question, we apply, herein, a high-level *ab initio* VB method. Two main issues are addressed in this article: quantifying the CSB character of MX, and dissecting the roles of the semicore fully filled (*n*−1)d shells in M atoms and of the lone pairs on X atoms in the bonding mechanism.

## 2. Computational Details

The structures of the target molecules were optimized at the CCSD level of theory in conjunction with three different basis sets: def2-TZVP [36], cc-pVQZ [37], and aug-cc-pVQZ [37]. These basis sets differ in size but are also associated with different effective core potentials (ECPs) for the coinage metal atoms. In particular, the def2-TZVP basis set is used together with the Stuttgart quasi-relativistic ECP for M atoms [38], whereas the cc-pVQZ and aug-cc-pVQZ are associated with the Stuttgart fully relativistic ECPs for transition and coinage metal atoms [39]. The CCSD(T) level is used for reference bond dissociation energies (BDE) to compare with the VB values. All coupled-cluster calculations were carried out with the Gaussian 09 program [40].

Valence Bond single-point energy calculations were carried out using the def2-TZVP and cc-pVQZ basis sets with the geometries optimized in the corresponding basis set. In all VB calculations with the cc-pVQZ, the g and h basis functions were deleted from the basis set. This allowed essentially the same accuracy as using the full basis set but with a significantly lower computational cost. The VB calculations were performed at two levels: with the VB Self-Consistent-Field (VBSCF) [11,12,13,14] and the Breathing Orbital VB (BOVB) methods [41,42,43]. These methods belong to the classical VB approach, in which the active orbitals are constrained to remain “strictly localized” on a given atom—i.e., they are only allowed to expand on the functions of the basis set that are centered onto one specific atom. In the VBSCF method, a common set of orbitals is used for all VB structures, and both the VB orbital coefficients and the structural coefficients are optimized simultaneously to minimize the total energy. In classical VB, VBSCF is the counterpart of the Molecular Orbital-based MCSCF method, and it essentially incorporates static correlation. In contrast, the BOVB wave function allows different sets of orbitals for different VB structures, which also enables the inclusion of dynamical correlation. The weights of VB structures can be defined in several different ways [11], and in this work we employed the most common Chirgwin–Coulson definition of weights [44]. Because BOVB wave function optimization may become more difficult when very minor structures are included, a selection from the full set of structures is typically used at this level of theory. In this work, the BOVB set includes all structures displaying a Chirgwin–Coulson weight larger than 0.5% in the full-structure VBSCF calculation. All VB calculations were carried out with the XMVB 3.0 program [45,46].

## 3. Results and Discussion

Table 1 shows the M–X bond lengths obtained at the CCSD level, along with experimental values. All employed basis sets in combination with the CCSD method provide slightly longer bond lengths than the experimental ones. The cc-pVQZ and aug-cc-pVQZ basis sets produce a remarkably good agreement with the experimental bond equilibrium distances, showing the mean absolute error to be less than 0.02 Å. The CCSD/def2-TZVP results are also very close to the reference experimental values, with a mean absolute error of 0.02 Å and a maximal error of 0.06 Å in the series. The reasonably good accuracy of the calculated bond distances validates the choice of the basis set and associated ECPs.

**Figure 1:**
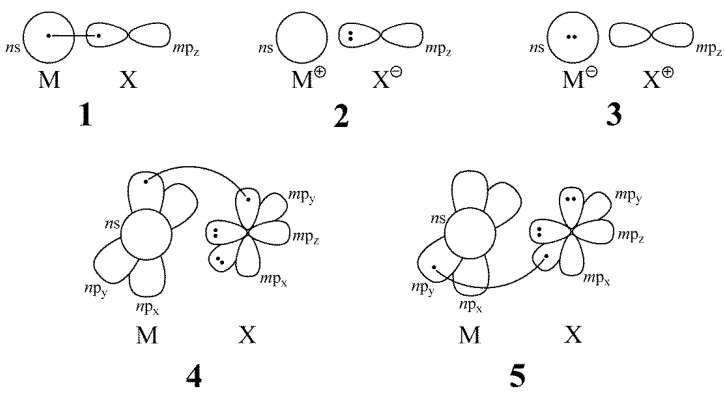
The selection of five VB structures describing the bonding in MX molecules (M = Cu, Ag, Au; X = F, Cl, Br).

VB structures are depicted in Figure 1. The valence shell of the metal atoms has an (*n*–1)d^10^ *n*s^1^ configuration, where *n* = 4, 5, and 6 for Cu, Ag, and Au, respectively. On the other hand, the halogen atoms have an *m*s^2^*m*p^5^ configuration, with *m* = 2, 3, and 4 for F, Cl, and Br, respectively. With the two bonded atoms oriented along the *z*-axis, VB structures **1**, **2**, and **3** describe an interaction between the *n*s orbital of the metal and the *m*p_z_ orbital of the halogen. A standard convention for drawing the VB structures was employed in Figure 1: the line connecting the two dots indicates covalent bonding, i.e., singlet coupling between the two electrons (represented by two dots) in the two active orbitals, with the corresponding (2c/2e) bond being completely described in Classical VB theory with the superposition of structures **1**–**3**.

We tested whether or not the filled (*n*–1)d^10^ sub-shell in the coinage metals can participate to some extent in the M-X bonding by extending the active set of orbitals and electrons to this shell. From a full structure VBSCF calculation explicitly including the d orbitals on the metal atom, it was found that all the additional structures (not represented here) that displayed a partially empty (*n*–1)d sub-shell on the metal atom have negligible weights, and their effect on the bond energies is marginal. We also tested the inclusion of VB structures that describe the dative π-type of bonding, arising from the donation of the lone pairs on X atoms (occupying *m*p_x_ and *m*p_y_ orbitals) into the vacant *n*p_x_ and *n*p_y_ orbitals on M. This effect is the opposite to the common “π backbonding”, as described in the standard Dewar–Chatt–Duncanson model [10,48,49], where the dative bond originates from a d-type electron pair on the metal. The full VB structure VBSCF calculations with the active space extended to the valence p shell revealed that only structures **4** and **5** in Figure 1 contributed significantly (weight > 0.5%) to this additional π-type bonding. Structures **4** and **5** could also be viewed as derived from the fundamental ionic structure **2** by transferring an electron from the doubly occupied *m*p_x_/*m*p_y_ orbital of the X atom to the corresponding virtual *n*p_x_/*n*p_y_ orbital on the M atom, thus leading to the neutral structures **4** and **5**. Computed VBSCF weights are provided in Table S3 of the Supplementary Information.

**Figure 2:**
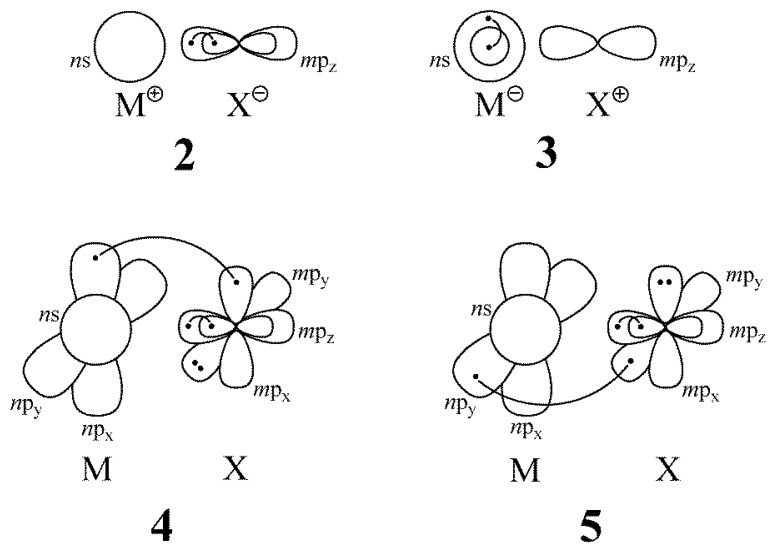
Schematic representation of the “splitting” of the *m*pz active orbital on X in structures **2**, **4,** and **5**, and of the *n*s orbital on M in structure **3** in the SD-BOVB level. The dots in these orbitals represent the electrons and the curve connecting the dots indicates a singlet coupling between the two electrons in the two split orbitals (M = Cu, Ag, Au; X = F, Cl, Br).

The VBSCF method is the standard classical VB method that includes static electron correlation only. In addition, the BOVB method includes part of the dynamic correlation that has been coined as the ‘‘Breathing Orbital effect” (BOE). The BOE has been demonstrated to be the main contributor to differential dynamical correlation—i.e., the component of dynamical correlation that varies significantly during a reaction process—and hence the BOE is the main source of improvement over VBSCF in terms of chemical accuracy where energy differences are concerned [43,50]. At the highest level of BOVB variants, known as the SD-BOVB level, two additional improvements are added compared to the standard BOVB level. First, the inactive electron pairs (doubly occupied orbital in all VB structures) are now allowed to delocalize (“D”) by spanning the whole basis of functions corresponding to their irreducible representation in the $C_{\infty v}$ point group. This basically reduces the Pauli repulsion between inactive pairs. Second, additional dynamic correlation is included by “splitting” (“S”) the active electron pairs into two coupled singly occupied orbitals. In our calculations, the doubly occupied *m*p_z_ orbital on X in structures **2**, **4** and **5**, and the *n*s orbital on M in structure **3**, were described by coupled pairs of orbitals, as schematically depicted in Figure 2. Hence, at this “S” level all active electron pairs are described as two-electron singlets coupled into a pair of orbitals, with the two orbitals of the coupled pair being centered either on different atoms in the case of a covalent or on the same atoms in the case of an ionic configuration. Splitting the ionic pairs basically involves radial dynamical correlation into the active pair and is a way to mimic, through orbitals, the action of explicit correlation functions such as the electron–electron terms in a Jastrow factor [51,52]. Although such a component of dynamical correlation is of secondary importance as compared with the BOE as energy differences are concerned, this may, however, lead to several kcal/mol of improvements in the BDE of electron-rich molecules [12,50].

**Figure 3:**
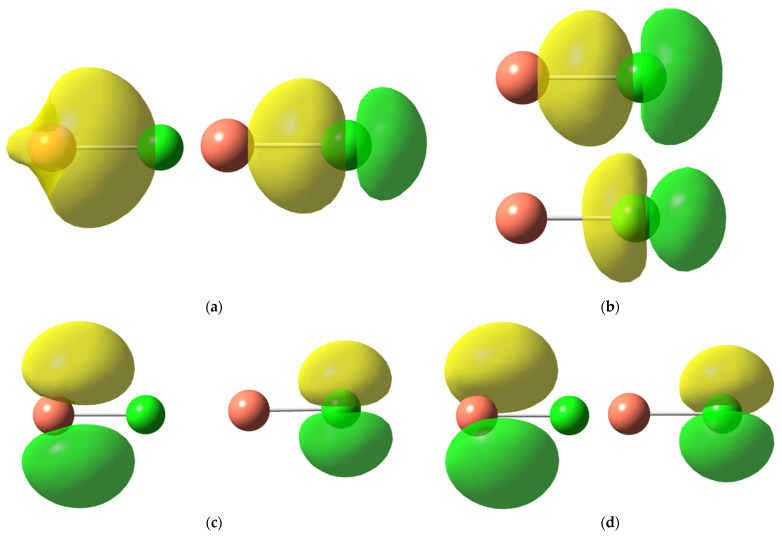
Active orbitals on Cu (left) and Cl (right) involved in the structures **1** (**a**), **2** (**b**), **4** (**c**), and **5** (**d**) obtained at the SD-BOVB/def2-TZVP level (isoval= 0.05).

It should be noted that, although we mark the VB orbitals as being of “s”, “p”, or “d” type for the sake of simplicity, our ab initio VB calculations enable the orbitals to expand over all types of basis functions and become polarized. More generally, VB orbitals expand on a subset of basis functions depending on the symmetry and potential localization constraints (see reference [12] for more details about practical VB calculations). As an illustrative example, the active orbitals obtained for CuCl are plotted in Figure 3. Figure 3b illustrates the split nature of the 3p_z_-type orbitals on Cl in the ionic structure **2** at the SD-BOVB/def2-TZVP level. One of these orbitals is more compact, the other one is more diffuse, and all of them are centered on a specific atom and allowed to polarize.

The BDE values obtained at the SD-BOVB level, along with the reference CCSD(T) and experimental values, are shown in Table 2. The experimental BDE values in different literature sources differ significantly, sometimes by more than 10 kcal/mol. Therefore, to assess the reliability of our VB wave functions, we also carried out CCSD(T) calculations using different basis sets. One can see that increasing the basis set size decreases the mean absolute errors (*MAE*) from 5.0 kcal/mol for the def2-TZVP basis to only 3.2 kcal/mol for the cc-pVQZ basis, relative to the reference CCSD(T)/aug-cc-pVQZ values. Furthermore, for a given basis set a good match is found between the SD-BOVB and CCSD(T) BDE values. Besides, if one compares the SD-BOVB and CCSD(T) BDE values obtained with the two basis sets, it can be seen that the SD-BOVB results converge more swiftly with the increasing basis set size. In the case of the cc-pVQZ basis, the SD-BOVB method slightly overperformed the CCSD(T) method, and gave results that, in many cases, were very similar to those obtained with CCSD(T) using the more extended aug-cc-pVQZ basis set. Hence, the comparison with the coupled-cluster results showed that the SD-BOVB wave function includes all the necessary physics to quantitatively describe such demanding systems. In what follows, the bonding nature in the examined molecules is analyzed based on the SD-BOVB/def2-TZVP wavefunctions.

As already mentioned, the VBSCF method is able to account for static electron correlation only, while the highest SD-BOVB level incorporates most of the differential dynamic correlation (ddc). CSB is usually typified by a large ddc, which in the prototype CSB molecule $F_{2}$ is larger than 30 kcal/mol [56]. The studied MX bonds displayed rather large ddc values ranging from 14 to 25 kcal/mol (Table S1 in Supplementary Materials), which may be indicative of the significant Charge-Shift Bonding character. This character can be directly and straightforwardly quantified through the “Charge-Shift resonance energy” (${RE}_{CS}$*)* within classical VB theory. In the case of 2c/2e bonded systems, represented by mixing of covalent and ionic structures, the ${RE}_{CS}$ is defined as the difference between the energies of the full VB structure wavefunction and the wavefunction consisting of the most stable VB structure (either the covalent or one of the ionic structures). The *%*${RE}_{CS}$ gives the percentage contribution of ${RE}_{CS}$ relative to the calculated BDE. As originally proposed, a given bond can be classified as CSB if the *%*${RE}_{CS}$ exceeds 50%. F_2_ is a typical “complete” CSB, as its *%*${RE}_{CS}$ is larger than 100% since its covalent structure is repulsive [21]. 

**Figure 4:**
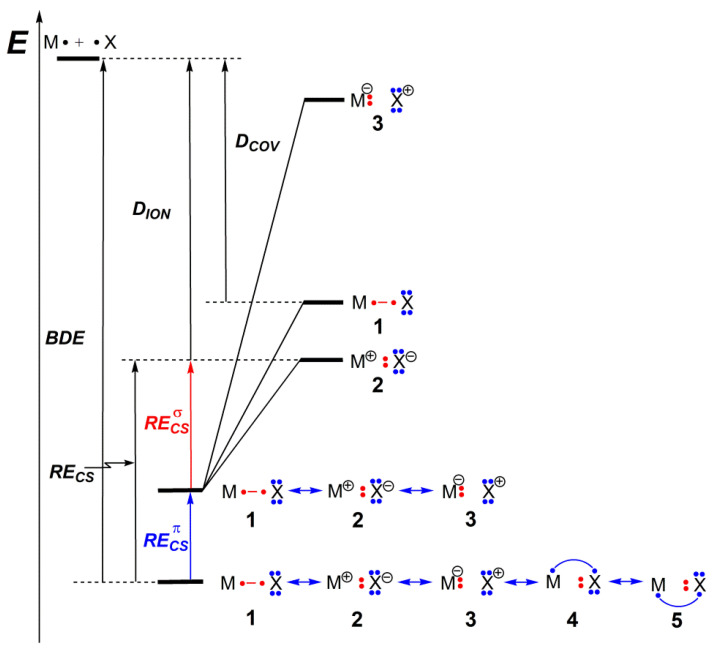
Schematic representation of VB interaction for MX molecules, involving the set of five VB structures displayed in Figure 1.

Within the studied series of molecules, structures **1**–**3** describe the *σ-*bonding as a typical 2c/2e bond. The VB interaction diagram displayed in Figure 4 depicts the case when the ionic structure **2** is the most stable one, which is the case for all dimers except AuCl and AuBr (Table S2 in Supplementary Materials). A similar diagram can be drawn in cases where **1** is dominant (Figure S1 in Supplementary Materials). The ${RE}_{CS}^{\sigma }$ was calculated with respect to the most stable of these three fundamental structures and was separately computed. As has been stated before, structures **4** and **5** can be interpreted as deriving from structure **2** by the transfer of one electron from a *m*p lone pair of the halogen atom to an empty *n*p orbital of the metal, thus allowing the inclusion of dative π-bonding. One should recall that dative bonds have been shown to be generally charge-shift bonds, as a major part of the bonding energy comes from the superposition of the corresponding VB structures [21]. As can be seen from Figure 4, the contribution of the dative π-bonding to the total BDE could be assessed through the term coined as ${RE}_{CS}^{\pi }$, corresponding to the stabilization of the BOVB wave function due to the mixing of the structures **4** and **5** with **1**–**3**.

As such, the total ${RE}_{CS}$ for these MX systems can be decomposed into σ and π components, according to Equation (1):

(1) $${RE}_{CS}{=RE}_{CS}^{\sigma }{+RE}_{CS}^{\pi }$$

Here, ${RE}_{CS}^{\sigma }$ is the “charge-shift” component of the σ bonding, while ${RE}_{CS}^{\pi }$ quantifies the dative π-bonding contribution.

The calculated ${RE}_{CS}$ value and its individual ${RE}_{CS}^{\sigma }$ and ${RE}_{CS}^{\pi }$ components, along with the ${\%RE}_{CS}$ relative to the total BDE, are shown in Table 3. For a given metal atom, the ${RE}_{CS}$ decreases down the halogen column significantly from MF to MCl and only slightly from MCl to MBr. This trend is expected, as the CS bonding mechanism is favored when highly electronegative atoms bearing contracted lone pairs (thus leading to large Pauli pressure) are involved. The fluorine atom is therefore the perfect candidate for CSB, and the other halogens also to a lesser and lesser degree as going down the column. Now, for a given halogen, the ${RE}_{CS}$ on the contrary *increases* down the coinage metal column. This originates from a decreasing M vs. X electronegativity difference, leading to a decreasing energy difference between structures **1** and **2** (Table S2 in Supplementary Materials), and thus to a larger resonance between the main two structures.

All in all, all the examined M-X bonds exhibited a significant CSB character, with substantially large ${RE}_{CS}$ ranging between 23 and 66 kcal/mol. Taking the ${\%RE}_{CS}$ values as a formal characterization of CSB, with the usual criterion that a *%RE* higher than 50% qualifies as a CSB, the CuX systems appeared to be dominantly polar-covalent with a significant CSB character. The AgX systems came out as borderline cases on its side. The ${\%RE}_{CS}$ approached 100% for AuCl and AuBr, and even surpassed it for AuF, the gold dimers unambiguously qualifies as pure CSBs on their side.

The ${RE}_{CS}^{\pi }$ can be taken as a measure of the strength of π-type bonding in the studied coinage metal halides. It can be seen that the π-bonding had a significant contribution (from 10 to 20%) to the total bonding energy. While the ${RE}_{CS}^{\sigma }$ exhibited very similar trends to those found for the total ${RE}_{CS}$, the ${RE}_{CS}^{\pi }$ displayed a somewhat different behavior. For each metal atom M, the ${RE}_{CS}^{\pi }$ decreased in the series from MF to MBr, indicating that F is the most efficient π-electron donor. However, for a given halogen, and contrary to the total ${RE}_{CS}$, the ${RE}_{CS}^{\pi }$ *decreased* down the coinage metal column. We can therefore conclude that the complete Charge-Shift Bond character of gold dimers is directly related to the sigma bond, and goes along with a reduction in the π-type component of bonding.

## 4. Conclusions

The detailed nature of bonding in coinage metal halide dimers has been dissected based on the high-level *ab initio* classical Valence Bond BOVB method. This method has been shown to provide accurate estimates of bond dissociation energies for these dimers, as compared with CCSD(T) combined with the large aug-cc-pVQZ basis set, and therefore the bonding analysis inferred from the BOVB wave function can be considered as fully reliable and quantitative. The main finding is that, similar to what has been found previously for coinage metal dimers themselves, [6] the sigma bond in gold halide dimers is a complete Charge-Shift Bond (CSB), while copper and silver halide dimers appear respectively as polar-covalent with substantial CSB character and borderline cases. This CSB character originates from the “Pauli pressure” arising from the interaction between the halogen pairs and the filled semicore (*n*−1)d pairs of the metal. This effect is magnified in the case of gold dimers because of the much larger relativistic-related shrinking of the valence *n*s and the expansion of the inner (*n*−1)d shells, both leading to larger repulsions with the halogen lone pairs and therefore creating a jump in the CSB character. A secondary finding of our analysis is that all dimers display a secondary though significant π bonding character, which in VB reading is associated with extra VB structures describing a dative π bond created from the donation of an *m*p lone pair of the halogen atom into a vacant *n*p valence orbital of the metal. Interestingly, this π-type component of bonding decreases with the increased CSB character of the sigma bond. All these findings could be elegantly summarized and visualized from the VB interaction diagram displayed in Figure 4.

Therefore, we have provided here extra confirmation that Lewis-related concepts such as chemical structures and the electron pair bond, and the classification of the latter in terms of covalent, ionic, and Charge-Shift categories, may be relevant not only in main group chemistry but also in transition metal complexes. The application of such concepts in the context of coinage metal-halide bonds is useful because, as discussed recently [21], the nature of the bond (covalent, ionic, charge-shift) is connected to the properties and reactivity of molecules. Last, the charge shift resonance energy can in principle be determined from experimental barrier difference data [21], so the application of this principle to coinage halides may potentially link this work to the experimental characterization of the resonance energy of bonds.

## Figures and Tables

**Table 1:** Optimized bond lengths of the MX molecules obtained at the CCSD level in combination with different basis sets. The mean absolute error (*MAE*) and the maximal observed absolute error (${AE}_{\max}$) were calculated with respect to the corresponding experimental values (italic numbers).

MX	def2-TZVP	cc-pVQZ	aug-cc-pVQZ	Exp. [47]
CuF	1.768	1.750	1.748	1.745
CuCl	2.103	2.068	2.062	2.051
CuBr	2.231	2.182	2.177	2.170
AgF	1.988	1.981	1.981	1.980
AgCl	2.295	2.294	2.288	2.281
AgBr	2.415	2.400	2.393	2.390
AuF	1.919	1.926	1.926	1.918
AuCl	2.214	2.220	2.216	2.199
AuBr	2.333	2.329	2.326	2.320
*MAE*	*0.02*	*0.01*	*0.01*	
${AE}_{\max}$	*0.06*	*0.02*	*0.02*	

**Table 2:** Bond dissociation energies (in kcal/mol) obtained at the SD-BOVB and CCSD(T) levels, together with experimental values for reference. The mean absolute errors (*MAE*) are calculated by reference to the corresponding CCSD(T) values in the largest basis set (italic), and for SD-BOVB also with respect to CCSD(T) values in the same basis set (in parenthesis and italic).

	def2-TZVP		cc-pVQZ		aug-cc-pVQZ	Exp.
	CCSD(T)	SD-BOVB	CCSD(T)	SD-BOVB	CCSD(T)	
CuF	91.8	90.4 ^a^	95.7	97.7 ^a^	99.0	98.9 [53]; 102.9 [54]
CuCl	83.2	82.1 ^a^	85.8	85.0 ^a^	89.0	90.3 [53]; 90.6 [54]
CuBr	77.3	74.9 ^a^	79.6	77.9 ^a^	83.3	79.1 [53]
AgF	76.4	73.2 ^a^	77.8	81.6 ^a^	81.1	85.3 [53]; 83.9 [54]
AgCl	71.8	70.8 ^a^	72.0	75.4 ^a^	74.7	66.7 [53]; 73.6 [54]
AgBr	67.5	64.8	67.3	70.5	70.8	67.0 [53]; 71.5 [54]
AuF	63.9	59.7	66.8	70.7	69.1	70.3 [53]; 73.8 [54]
AuCl	64.0	61.0	65.5	63.1 ^a^	68.2	66.9 [53]; 72.2 [55]
AuBr	60.9	56.5	62.0	64.2	66.3	50.9 [53]; 68.3 [55]
*MAE*	*5.0*	*7.6 (2.6)*	*3.2*	*1.8 (1.5)*	*0.00*	

^a^ Structure **3** was excluded in these calculations due to the low weight (<0.5%) at the VBSCF level.

**Table 3:** Charge-Shift Resonance Energy (${RE}_{CS}$, in kcal/mol) and its components depicted in Figure 4 obtained at the SD-BOVB/def2-TZVP level. The percentage of charge-shift resonance energy relative to the individual dimer bond dissociation energy is also shown (italic numbers).

**MX**	${RE}_{CS}^{\sigma }$	${\%RE}_{CS}^{\sigma }\;$	${RE}_{CS}^{\pi }\;$	${\%RE}_{CS}^{\pi }\;$	${RE}_{CS}\;$	${\%RE}_{CS}$
CuF	21.8	*24.1*	16.9	*18.7*	38.7	*42.8*
CuCl	15.6	*19.0*	9.5	*11.6*	25.2	*30.6*
CuBr	15.0	*20.0*	7.9	*10.5*	22.9	*30.5*
AgF	28.2	*38.5*	12.5	*17.0*	40.7	*55.5*
AgCl	26.8	*37.8*	8.3	*11.7*	35.1	*49.5*
AgBr	27.3	*42.1*	6.4	*9.8*	33.7	*51.9*
AuF	55.4	*92.8*	10.1	*17.0*	65.5	*109.8*
AuCl	47.4	*77.8*	7.2	*11.9*	54.6	*89.7*
AuBr	44.3	*78.5*	6.1	*10.9*	50.4	*89.4*

## Data Availability

XMVB output files corresponding to this study may be provided, if necessary, upon request to the corresponding authors.

## Supplementary Materials

The following supporting information can be downloaded online. Table S1: BDE values (in kcal/mol) obtained at the SD-BOVB/cc-pVQZ level and the dynamical correlation (ddc, in kcal/mol) effect defined as the difference between the BDE values obtained at the VBSCF and SD-BOVB levels with the cc-pVQZ basis set. Table S2: The Chirgwin–Coulson weights (in %) of VB structures and the energy difference between the structures **1** and **2** ($E_{1}{-E}_{2}$) in kcal/mol, at the SD-BOVB level using the def2-TZVP basis set. Table S3: The Chirgwin–Coulson weights (in %) of VB structures at the VBSCF level using the def2-TZVP basis set. Figure S1: Schematic representation of VB interaction diagram for MX molecules showing the case when the covalent structure **1** is dominant.
