# Supplementary material for: On the Nature of the Bonding in Coinage Metal Halides
Source: Molecules. 2022 Jan 13;27(2):490. doi: 10.3390/molecules27020490 (PMC8780489; doi:10.3390/molecules27020490)
Supplement: Supplementary file 1 [file molecules-27-00490-s001.zip › molecules-1534263-supplementary.pdf]

Supplementary Material

On the Nature of the Bonding in Coinage Metal Halides

Slađana Đorđević [1], Slavko Radenković [1,*], Sason Shaik [2] and Benoît Braïda [3,*]

[1] Faculty of Science, University of Kragujevac, 34000 Kragujevac, Serbia; sladjadj93@gmail.com. ORCID: 0000-0003-0194-3927.
[2] Institute of Chemistry, Hebrew University of Jerusalem, 91904 Jerusalem, Israel; sason@yfaat.ch.huji.ac.il or sason.shaik@gmail.com. ORCID: 0000-0001-7643-9421
[3] Laboratoire de Chimie Théorique, Sorbonne Université, UMR7616 CNRS, 75005 Paris, France
* Correspondence: slavkoradenkovic@kg.ac.rs (S.R.), ORCID: 0000-0002-1928-5494; benoit.braida@sorbonne-universite.fr (B.B.), ORCID: 0000-0003-3725-3215.

The BDE obtained at the SD-BOVB/cc-pVQZ level, together with the differential dynamical correlation (ddc) values, obtained by taking the difference between SD-BOVB and VBSCF BDEs, are collected in Table S1. As expected, the largest ddc effect, between 23 and 25 kcal/mol, was found in the coinage-metal fluorides. All in all, the studied MX bonds show rather large ddc values ranging from 14 to 25 kcal/mol.

Table S1. BDE values (in kcal/mol) obtained at the SD-BOVB/cc-pVQZ level and the dynamical correlation (ddc, in kcal/mol) effect defined as the difference between the BDE values obtained at the VBSCF and SD-BOVB levels with the cc-pVQZ basis set. The results in the parenthesis show the percentage contribution of ddc in the total BDE.

MX	BDE (SD-BOVB)	ddc
CuF	97.7	25.3 (26.5%)
CuCl	85.0	15.9 (15.9%)
CuBr	77.9	14.6 (14.6%)
AgF	81.6	23.0 (23.0%)
AgCl	75.4	15.4 (15.4%)
AgBr	70.5	15.0 (15.0%)
AuF	70.7	23.2 (23.2%)
AuCl	63.1	17.1 (17.1%)
AuBr	64.2	21.9 (21.9%)

Table S2 display the Chirgwin-Coulson weights obtained at the SD-BOVB/def2-TZVP level (first five columns), together with the energy difference between the structures **1** and **2** with each of them computed in two separate one-structure calculations (last column).

The E_1-E_2 quantity for each MX bond decreases monotonically in the series F-Cl-Br, indicating that the covalent structure **1** gets closer to the ionic structure **2**, the latter remaining the lowest of the two except in the AuCl and AuBr cases, in accordance with the decreasing electronegativity difference between a given metal and the halogen atoms in the F-Cl-Br series. Similarly, the E_1-E_2 difference appears to also logically decrease in the Cu-Ag-Au series for a given halogen atom.

The VB structure weights do not appear to provide a similar clear trend such as the E_1-E_2 energy difference. This is because the weight balance arising after the structure mixing depends not only on the individual separate structure energies, but also on the resonance interaction between them. When the resonance effect is large, as it is the case in CSBs, the outcoming weights may not be a reliable of the ranking in energy of the separate individual structures. Even further, in case of CSBs the bonding is dominated by RE_{CS}, and thus in the covalent vs. ionic relative weights are not predictive of the nature of the bond (see ref. 15 in the main text).

Table S2. The Chirgwin-Coulson weights (in %) of VB structures (first five columns, italic numbers) and the energy difference between the structures **1** and **2** (E_1-E_2) in kcal/mol (last column, regular numbers), at the SD-BOVB level using the def2-TZVP basis set. The sign "-" indicates that the respective VB structure was not included in the given calculation.

MX	1	2	3	4	5	E_1-E_2
CuF	*38.71*	*42.47*	-	*9.41*	*9.41*	78.07
CuCl	*47.58*	*36.42*	-	*8.00*	*8.00*	31.31
CuBr	*50.29*	*35.35*	-	*7.18*	*7.18*	23.10
AgF	*36.35*	*49.31*	-	*7.17*	*7.17*	55.70
AgCl	*44.60*	*41.30*	-	*7.05*	*7.05*	17.93
AgBr	*39.60*	*42.29*	*4.81*	*6.65*	*6.65*	10.55

AuF	*35.07*	*51.90*	*2.01*	*5.51*	*5.51*	19.38
AuCl	*36.75*	*47.11*	*4.54*	*5.79*	*5.79*	−12.13
AuBr	*39.11*	*44.20*	*5.63*	*5.53*	*5.53*	−17.22

The Table S3 below displays VBSCF weights obtained after wave-function optimization when structures **4** and **5** are included or not included in the selection. Comparing the two cases reveals that the weight of the covalent structure **1** barely changes whether **4–5** are included or not. Comparison between tables S2 and S3 shows that for some of the molecules (AgBr and Au-halides) the SD-BOVB weight of the covalent structure is significantly smaller than the one obtained at the VBSCF level. This is a common feature of the BOVB method, which, because specific set of orbitals are optimized for each VB structure, allows a more balanced description of covalent and minor ionic structures in comparison to the VBSCF method.

Table S3. The Chirgwin-Coulson weights (in %) of VB structures at the VBSCF level using the def2-TZVP basis set. The sign "-" indicates that the respective VB structure was not included in the given calculation.

**MX**	**1**	**2**	**3**	**4**	**5**
CuF	36.06	63.94	-	-	-
	37.82	41.44	-	10.37	10.37
CuCl	45.54	54.46	-	-	-
	45.82	36.96	-	8.61	8.61
CuBr	49.05	50.95	-	-	-
	49.08	34.99	-	7.97	7.97
AgF	35.26	64.74	-	-	-
	37.22	47.54	-	7.62	7.62
AgCl	44.20	55.80	-	-	-
	44.16	41.36	-	7.24	7.24
AgBr	47.83	51.90	0.27	-	-
	48.11	36.68	0.92	7.15	7.15
AuF	45.04	54.83	0.13	-	-
	45.17	41.28	0.50	6.53	6.53
AuCl	48.76	49.12	2.12	-	-
	47.30	37.71	2.28	6.35	6.35
AuBr	50.52	46.75	2.73	-	-
	49.31	35.26	2.96	6.24	6.24

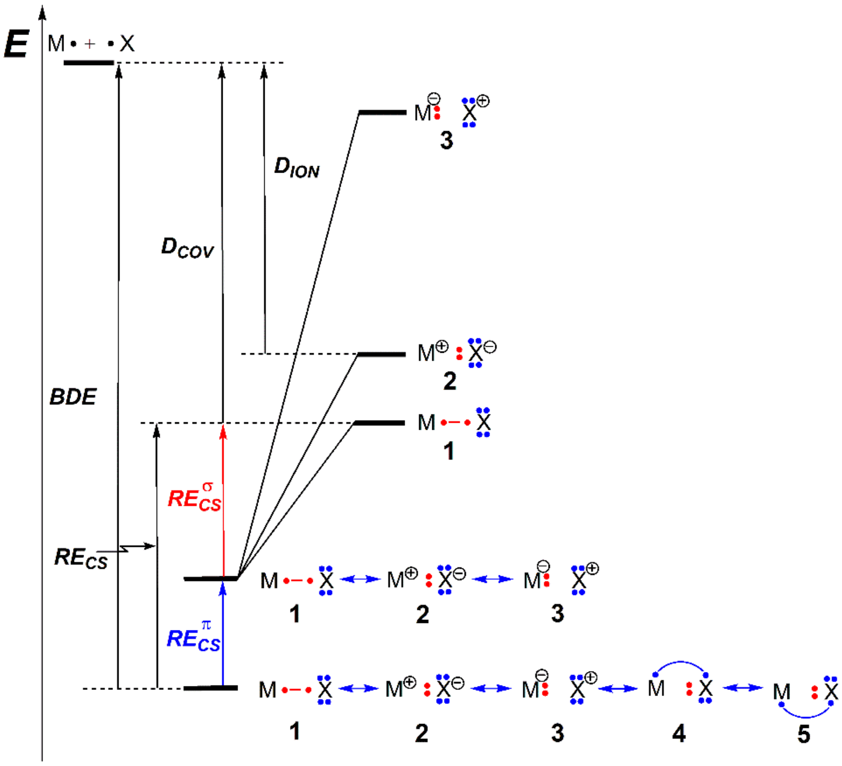

Figure S1. Schematic representation of VB interaction diagram for MX molecules showing the case when the covalent structure **1** is dominant.
